# Cigarette smoke enhances initiation and progression of lung cancer by mutating Notch1/2 and dysregulating downstream signaling molecules

**DOI:** 10.18632/oncotarget.22924

**Published:** 2017-11-25

**Authors:** Wei Li, Jihong Zhou, Yuqing Chen, Gengyan Zhang, Peng Jiang, Lei Hong, Yuangbing Shen, Xiaojing Wang, Xiaomeng Gong

**Affiliations:** ^1^ Department of Respiratory Disease, The First Affiliated Hospital of Bengbu Medical College, Bengbu 233004, China; ^2^ Provincial Key Laboratory of Respiratory Disease in Anhui, Bengbu 233004, China; ^3^ Department of Biochemistry and Molecular Biology, Bengbu Medical College, Bengbu 233004, China; ^4^ Department of Pathology, The First Affiliated Hospital of Bengbu Medical College, Bengbu 233004, China

**Keywords:** lung cancer, Notch1, Notch 2, mutations, signaling

## Abstract

Lung cancer is the primary cause of cancer related deaths in the western world and smoking significantly increases the risk of developing lung cancer. Smoking enhances lung cancer initiation and progression. The effects of cigarette smoke on lung cancer are mediated by the presence of highly mutagenic substances, including nicotine, leading to mutations in oncogenes and tumor suppressor genes. An emerging pathway in cancer is the Notch signaling pathway which is essential for embryonic lung development and tissue homeostasis. The role of Notch signaling in lung cancer remains controversial and no studies have directly linked cigarette exposure to mutations in Notch. Therefore, we investigated the direct effect of Notch signaling pathways on cigarette-induced lung tumors and the correlation between smoking and mutations in Notch leading to altered downstream signaling. Human cell lines, mouse models and clinical lung cancer samples were utilized in this study. Cigarette-induced *in vitro* human lung cancer models and *in vivo* mouse models demonstrated strong effects of cigarette exposure on the Notch signaling pathway. Immunohistochemistry (IHC) of 50 clinical samples collected from smokers and non-smokers with and without lung cancer also demonstrated a link between smoking and changes in Notch signaling. Finally, 34 lung cancer samples analyzed through direct sequencing indicated smoking significantly increased small nucleotide polymorphisms (SNPs) in Notch 1 and 2 and specific SNPs significantly modulated expression levels of downstream signaling pathway molecules. Taken together, these results demonstrate a direct effect of smoking on the Notch signaling pathway leading to lung cancer initiation and progression.

## INTRODUCTION

The principal cause of cancer related deaths in the western world is lung cancer. Smoking significantly increases the risk of developing numerous tumor types, but none more so than lung cancers. Along with the effects of smoking on lung cancer initiation, which have been appreciated for over 50 years, tobacco use has also been linked to lung cancer progression and aggressiveness [1]. Although the correlative links between smoking and lung cancer were known since the 1930s it was not until the 1980s that scientist determined that carcinogens in tobacco smoke induced mutations in specific oncogenes and tumor suppressor genes leading to the initiation and progression of lung cancer [2]. High rates of mutations caused by smoking are observed in the prevalent oncogene, KRAS, as well as the tumor suppressor gene, p53 [2, 3], although it is clear through clinical and experimental evidence that other genes are mutated as well.

Recently, numerous pathways involved in embryonic development have been linked to lung cancer. Specifically, members of the Notch transmembrane receptor family and downstream signaling molecules are dysregulated during lung cancer progression [4, 5]. There are 4 Notch receptors that function as heterodimeric receptors containing an extracellular N-terminal domain bound to transmembrane and C-terminal segments. Notch signaling is essential for embryogenesis, including lung development, and functions in post-natal tissue homeostasis as well [4]. The role of Notch receptors in non-small cell lung cancer (NSCLC) remain controversial, with studies indicating both tumor suppressive and supportive roles [6, 7]. Indeed, different Notch family members may have tumor supporting and tumor suppressing roles, such as Notch1 and Notch2, respectively [8]. It is becoming clear that the role of Notch singling in lung development and lung cancer is highly dependent on the cellular and environmental context being investigated [9–17].

Notch activating mutations have been detected in in patients with NSCLC which correlated with a worse prognosis in a subset of patients [18]. With mounting evidence indicating the role of Notch signaling in lung cancer we set out to determine the direct effects of cigarette smoking on Notch signaling and whether these effects were mediated by mutations in the Notch gene.

## RESULTS AND DISCUSSION

### Development and characterizaiton of *in vitro* and *in vivo* lung cancer models induced by cigarette byproduct exposure

**Figure 1:**
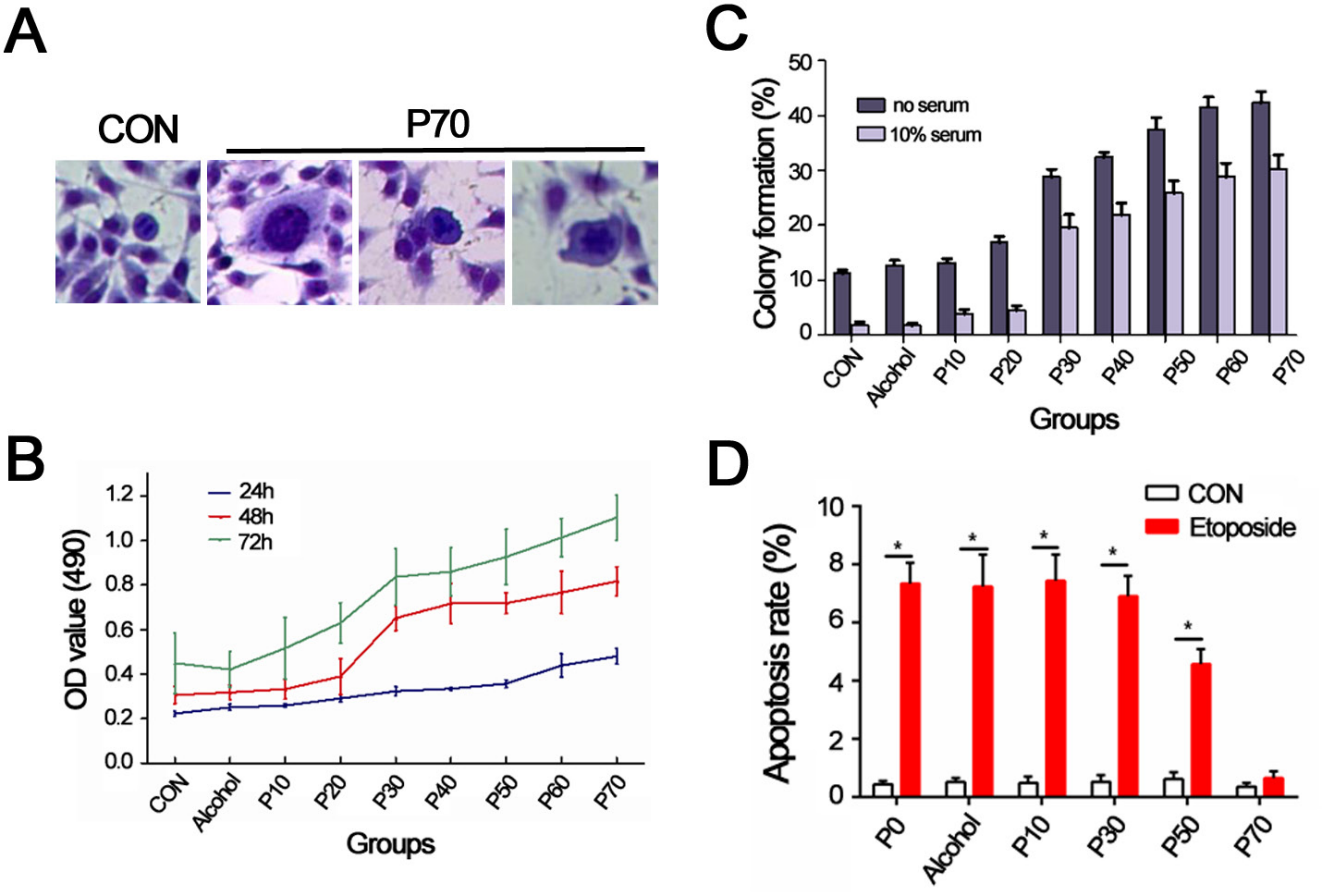
Establishment of BEP2D cell model of lung cancer induced by cigarette smoke condensates **(A)** Giemsa staining for control and BEP2D cells induced with CSCs at 70^th^ passage (P70, 200×). P70 cells show increase of nucleus to cytoplasm ratio and pathological mitosis. **(B)** MTT analysis for control and BEP2D cells induced by CSC at different generations. **(C)** Colony formation for control and BEP2D cells induced by CSC at different generations. **(D)** Apoptosis rate for control and BEP2D cells induced by CSC at different generations. Data are plotted as mean ± SD at least three independent experiments, ^*^P<0.05, compared to control group.

To determine whether smoking in humans directly induces mutations in Notch it was essentail to generate a lung cancer model caused by cigarette by products. This was achieved by culturing human bronchial epithelial cells (BEP2D) in serum free LHC-8 medium supplemented with CSC at a concentration of 1 cigarette/ml for an extended period of time. Phenotypic histological analysis of BEP2D cells cultured with CSC demonstrated an altered phenotype associated with increased nucleus to cytoplasm ratio and pathological mitosis at passage 70 compared to control untreated BEP2D cells (Figure 1A). CSC treatment also led to higher rates of proliferation in BEP2D cells, especially at later passage numbers (Figure 1B). Additionally, higher rates of proliferation were observed in BEP2D cells treated with CSC for 48 and 72 hours (Figure 1B, red and green line, respectively) compared to cells treated for only 24 hours (Figure 1B, blue line). An excellent measure of cancer cell tumorigenecity, agressiveness and “stemness” is colony formation in soft agar [19]. A dramatic increase in the ability of BEP2D cells to form colonies was observed in cells treated with CSC after 30 passages in both serum-free and 10% serum conditions. Conversly, the succeptability of CSC treated BEP2D cells to etoposide induced apoptosis signiicantly decreased over time (Figure 1D). Indeed, no difference in the percentage of apoptosis is observed in etoposide treated CSC-induced BEP2D cells and control CSC-induced BEP2D cells at passage 70, indicating that these cells were completely resistant to etoposide (Figure 1D). These results indicate that CSC treated BEP2D cells provides an excellent *in vitro* model to study cigarette-induced lung cancer initaiton and progression.

**Figure 2:**
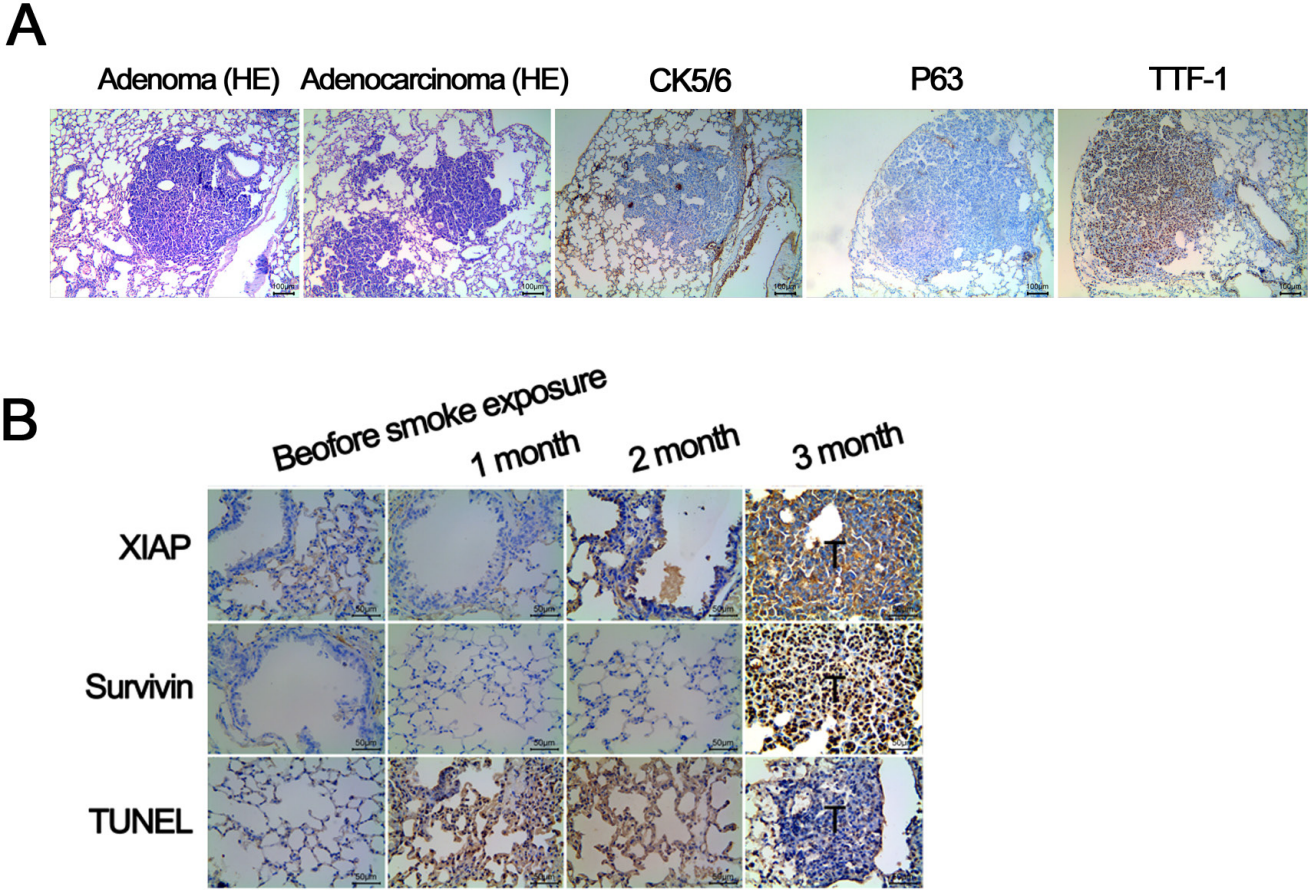
Validation of A/J mouse lung cancer model induced by smoke exposure **(A)** All of the A/J mice form lung tumors 9 months after smoke exposure. H&E staining shows: 2 cases of adenoma, 8 cases of adenocarcinoma; immunohistochemical study for 8 cases of adenocarcinoma show: TTF - 1 was strong positive, P63 and CK56 were negative, confirming the finding of adenocarcinoma. **(B)** Compared with control lung tissue, the expression of antiapoptotic proteins XIAP and Survivin in mice with lung cancer is significantly higher (P < 0.01). TUNEL staining demosntrates the apoptosis rate of tumor tissue is significantly lower compared with lung tissue of control mice (P < 0.01).

Next, an *in vivo* model was generated to supplement our *in vitro* cigarette-induced lung cancer. All A/J mice exposed to 7 mg/m^3^ of nicotine for 50 minutes a day 6 days a week developed lung cancer after 9 months. Out of 10 mice, 2 developed adenomas and 8 developed adenocarcinomas (representative H&E, Figure 2A). All adenocarcinoma cases stained positive for Transcription Termination Factor 1 (TTF-1) and negative for P63 and CK16 (Figure 2A). A significant increase in expression of anti-apoptotic proteins, X-linked inhibitor of apoptosis (XIAP) and Survivin, corresponded with a significant decrease in terminal deoxynucleotidyl transferase dUTP nick end labeling (TUNEL) levels after 3 months in nicotine exposed mice compared to control untreated animals (Figure 2B). Taken together, these results indicate that A/J mice exposed to nicotine develop adenomas/adenocarcinomas associated with decreased levels of apoptosis, providing an *in vivo* model for lung cancer induced by cigarette exposure.

### Expression of Notch pathway members is dysregulated in *in vitro* and *in vivo* cigarette-induced lung cancer models

**Figure 3:**
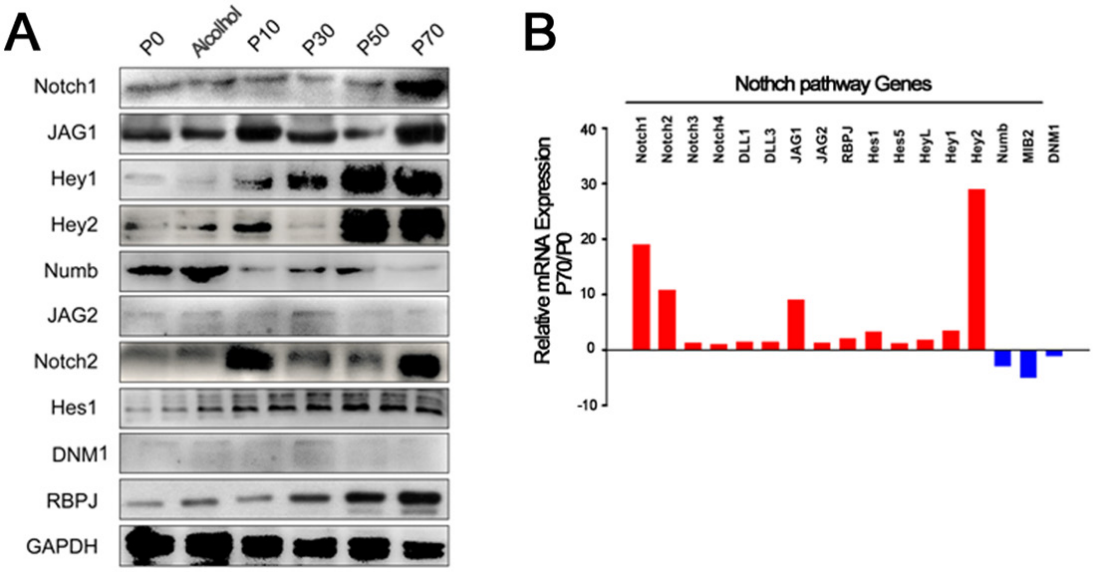
Expression of Notch pathway members in BEP2D cells induced by CSC at different passage numbers **(A)** Western blots for protein expression of NOTCH signlaing pathway members, including NOTCH1, JAG1, Hey1, Hey2, Numb, JAG2, NOTCH2, Hes1, DNM1, RBPJ, in BEP2D cells from passage 0 to passage 70. GAPDH was used as a loading control. **(B)** qPCR analysis for comparisons of NOTCH pathway gene expression in BEP2D cells at passage 70 versus passage 0.

Protein and ribonucleic acid (RNA) expression of Notch family members were assessed in CSC-treated BEP2D cells as well as nicotine supplemented A/J mice to determine whether the Notch signaling pathway was dysregulated in lung cancer associated with cigarette exposure. Notch family members included; Notch1, jagged1 (JAG1), hairy/Enhancer-Of-Split Related With YRPW Motif 1 (Hey1), hairy/Enhancer-Of-Split Related With YRPW Motif 2 (Hey2), Numb, jagged2 (JAG2), Notch2, hairy and enhancer of split 1 (Hes1), Dynamin-1 (DNM1) and recombining binding protein suppressor of hairless (RBPJ). Protein expression levels of Notch family members was assessed in CSC treated BEP2D cells through Western Blots with Glyceraldehyde 3-phosphate dehydrogenase (GAPDH) used as a loading control (Figure 3A). Proteins that were expressed at higher levels in BEP2D cells following prolonged exposure to CSC included Notch1, JAG1, Hey1, Hey 2, JAG2, Notch 2, Hes1 and RBPJ, whereas Numb expression in BEP2D cells decreased over time (Figure 3A). Similar findings were observed in the A/J mice exposed to nicotine for 9 months (data not shown). Gene expression analysis for Notch family members demosntrated relatively higher levels of Notch1, Notch2, JAG1 and Hey2 in CSC-treated BEP2D cells at passage 70 compared to untreated controls (Figure 3B). Other Notch family members were upreguatled to a lesser extent while Numb transcript expression was lower in the CSC-treated BEP2D cells compared to untreated (Figure 3B). Again, similar results were observed in A/J mice epxosed to nicotine (data not shown), indicating that the Notch pathway is highly dysregulated during lung cancer progression. In agreement with published studies, these results demonstrate a tumor supressive role of the Notch signaling pathway member, Numb, along with tumor supportive functions of other Notch signaling members [20, 21].

### Levels of Notch pathway genes are altered in normal and cancerous human bronchial mucosa of heavy smokers compared to never-smokers

**Figure 4:**
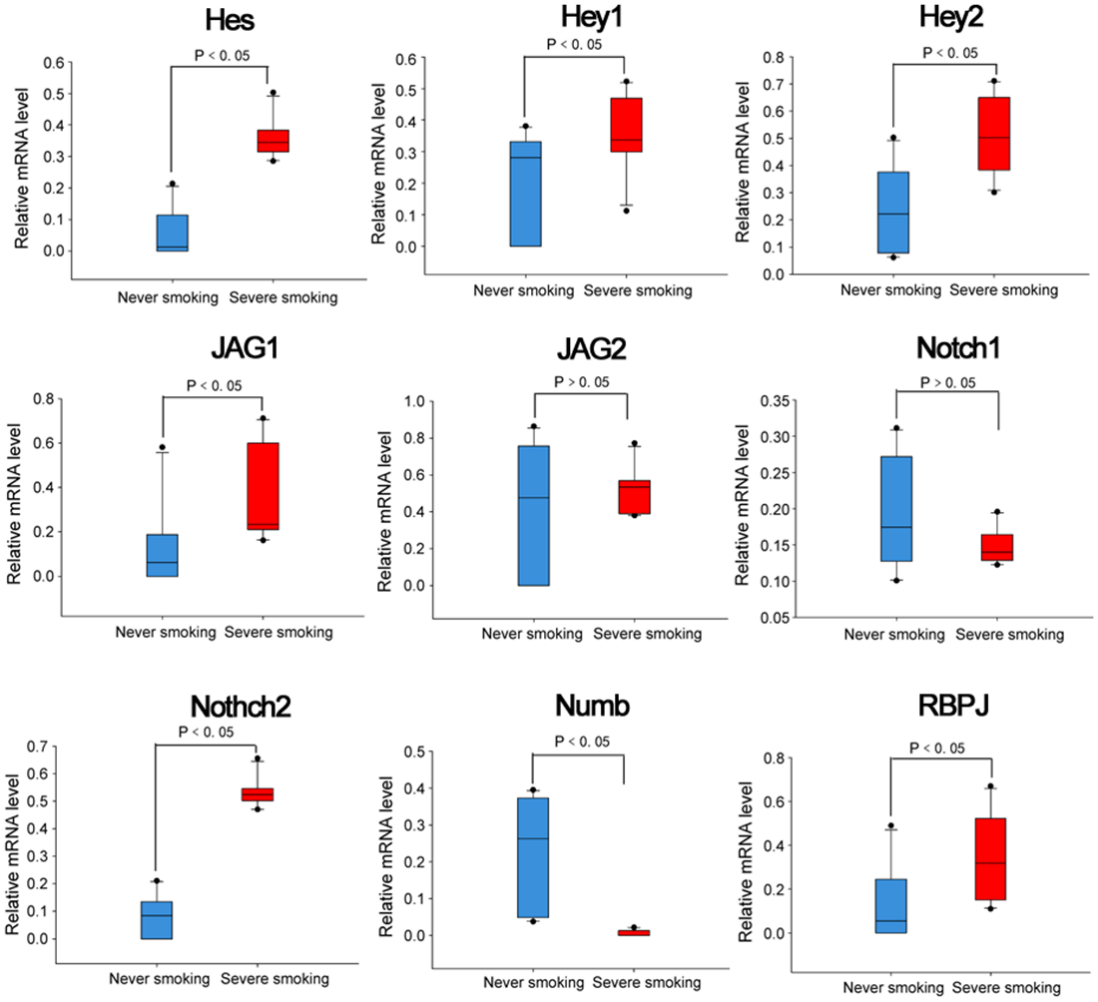
mRNA expression level of Notch pathway genes in bronchial mucosa of heavy smokers and never-smokers **(A)** qPCR analysis was perforemed to assess mRNA expression of NOTCH signlaing pathway members, including NOTCH1, JAG1, Hey1, Hey2, Numb, JAG2, NOTCH2, Hes, DNM1, RBPJ, in lung samples collected from heavy-smokers and non-smokers. GAPDH was used as an endogenous control.

**Figure 5:**
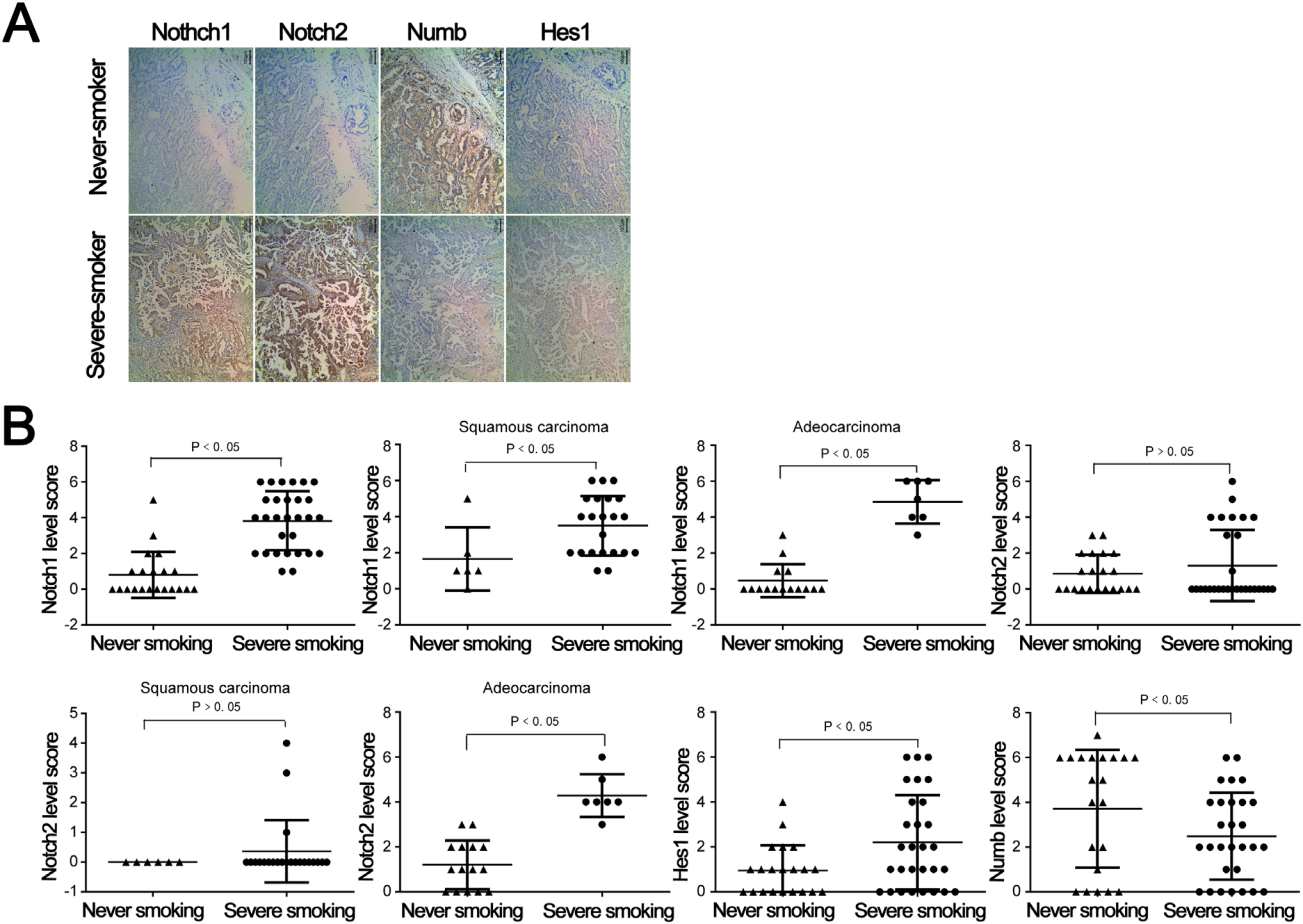
Expression of Notch pathway proteins in never smoking and severe smoking subgroup among NSCLC patients **(A)** IHC staining of paraffin specimens obtained from patients of the two subgroups. **(B)** Statistical plot of each Notch pathway member protein expression in the two subgroups.

Transcript leves of Notch signlaing pathway members, including NOTCH1, JAG1, Hey1, Hey2, Numb, JAG2, NOTCH2, Hes, DNM1, RBPJ, in samples collected from heavy smokers and non-smokers without lung cancer was assessed (Figure 4). Significant differences were observed in Hes, Hey1, Hey2, JAG1, Notch2, Numb and RBPJ (P<0.05) while no significant differences were observed in transcript levels of JAG2 and Notch1 between the heavy and non-smoking groups (Figure 4). Hes, Hey1, Hey2, JAG1, Notch2 and RBPJ were expressed at higher levels in smokers while Numb was expressed at lower levels. Next, samples collected from patients with NSCLC who were either heavy smokers or non-smokers were assessed for levels of Notch signaling pathway members. Representative immunohistochemistry (IHC) microphotgraphs are provided from patients with NSCLC who were either severe smokers or never smoked (Figure 5A). To highlihgt differences in Notch1 and 2 expression in different NSCLC subtypes, IHC scores for Notch1 and 2 levels in total NSCLC, squamous carcinoma or adenocarcimona are provided (Figure 5B). Signficant increases in Notch1 protein expression were observed in the severe smokers of both subgroups, while Notch2 epxression was only found to be significantly higher in smokeres of the adenocarcinoma subgroup. Hes1 IHC scores were also signficantly higher in smokers with NSCLC, although Numb expression was significantly lower in smokers with NSCLC (Figure 5B).

**Figure 6:**
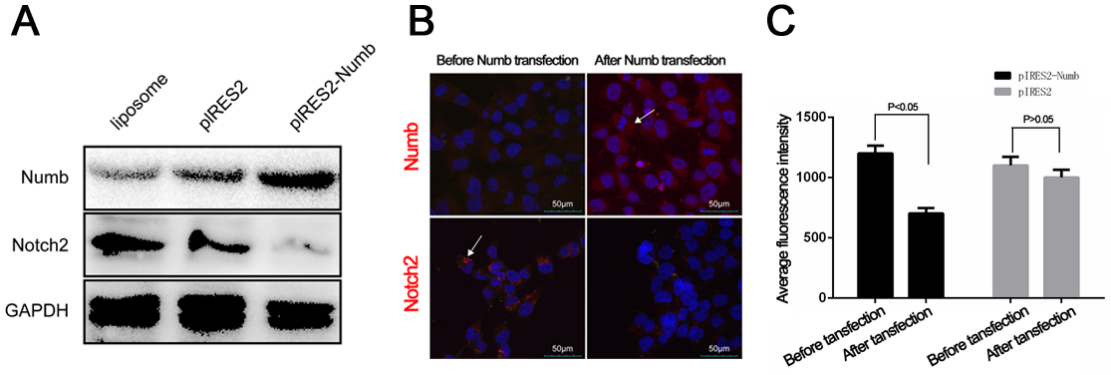
Numb overexpression significantly decreased expression of Notch2 protein in P70 cell **(A)** Western blot analysis for Notch and Numb in three groups, GAPDH is used as loading control. **(B)** Immunofluorescence test for Notch2 before and after Numb transfection in P70 cell. **(C)** Relative fluorescence intensity values of Notch2 protein are plotted as mean ± SD.

Thus far, our data demosntrates increases in the expression of Hes, Hey1, Hey2, JAG1, Notch2 and RBPJ along with consistent decreases in the levels of Numb in samples exposed to cigarettes versus no exposure. Numb has been shown to be a negative regulator of Notch signaling, therefore, we tested whether induced expression of Numb modulated Notch expression. Using our CSC induced-lung cancer model cells at p70 that are induced to overexpress Numb display significantly lower levels of Notch2 protein as assessed by Western blots (Figure 6A) and IHC (Figure 6B and 6C). Consistent with our *in vitro* and *in vivo* studies, these clinical studies reveal a signicant alteration of Notch signaling associated with smoking in both normal lung samples and various categories of lung cancer samples.

### Smoking directly mutates the Notch genes leading to dysregulation of Notch signlaing pathway member, Hes1

**Figure 7:**
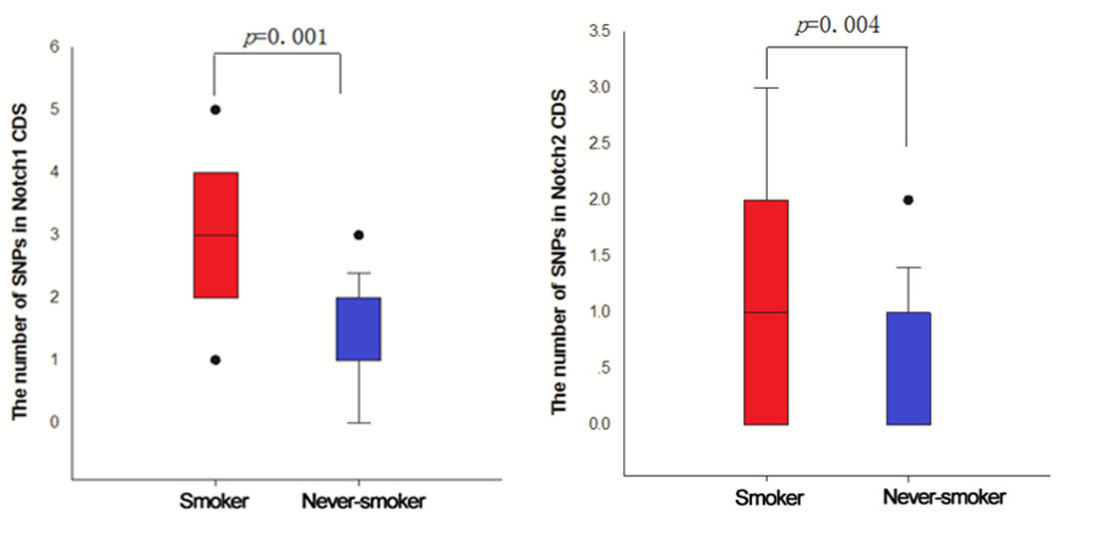
The number of SNPs in Notch1/2 CDS of smoker and never-smoker groups Direct sequencing of samples collected from smokers and non-smokers with NSCLC was performed to determine number of SNPs in NOTCH1 and 2 genes.

**Figure 8:**
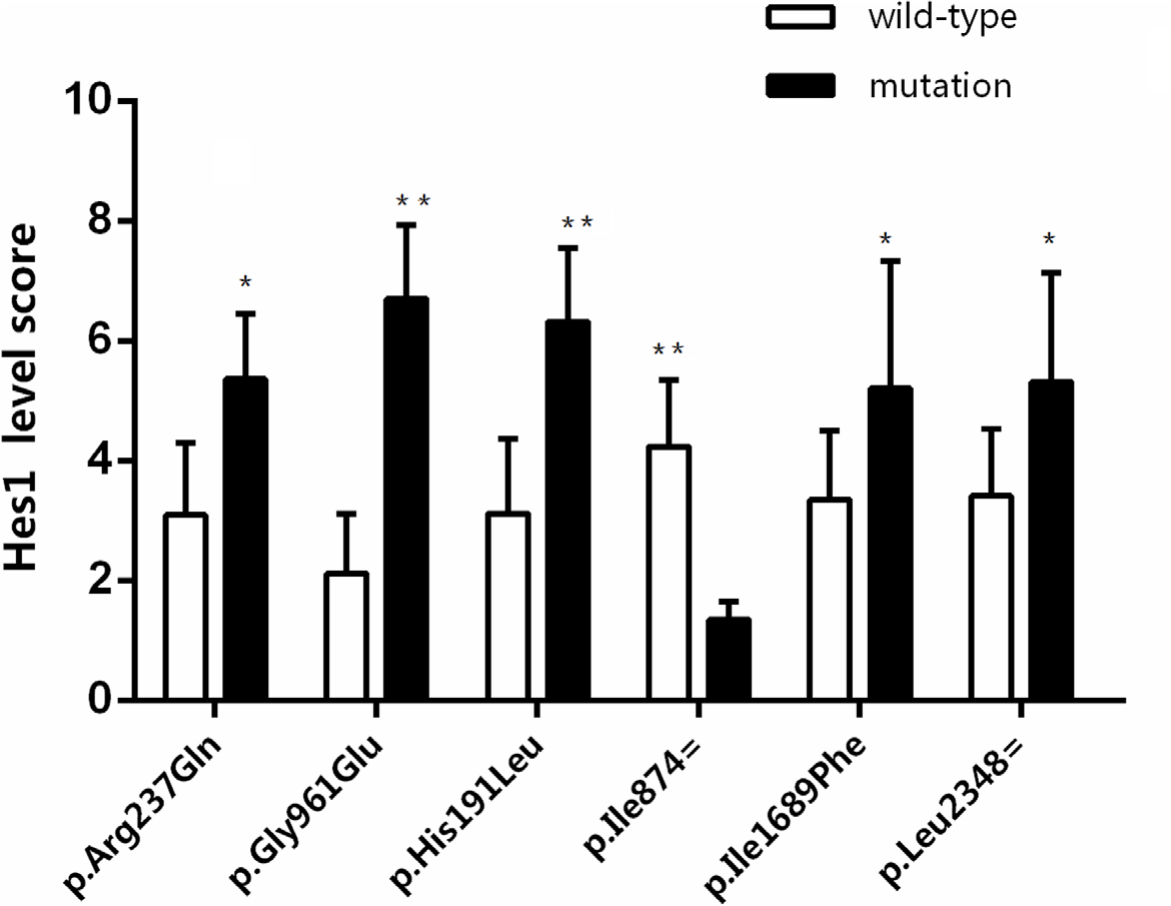
The effect of six SNPs on Hes1 expression level in cancer tissue of NSCLC by immunohistochemistry Immunnohistochemistry for Hes1 was performed on NSCLC samples from patients with specific SNPs and expression levels scored from 1-10. ^*^P<0.05, ^**^P<0.01 wild-type vs mutation.

Cigarette byproducts, especailly nicotine, are potent mutagens and have been demonstrated to mutate numerous oncogenes and tumor suppressor genes. Here, we assessed whether a correlation between smoking and mutations in the Notch genes exists utilzing direct sequencing of lung tumor samples from patients with NSCLC. A significant increase in the number of small nucleotide polymorphisms (SNPs) was observed in both the Notch1 gene (p=0.001) and the Notch 2 gene (p=0.004) (Figure 7). Next, we assessed whether specific SNPs in the Notch gene led to changes in Notch signaling pathway family members by assessing levels of Hes1 in tumor samlpes from NSCLC patients with mutated and non-mutated Notch genes. All SNPs tested, including p.Arg237Gln, p.Gly961Glu, P.His191Leu, P.Ille874=, p.Ile1689Phe and p.Leu2348=, demonstated significant differences in their Hes1 epxression scores compared to unmutated controls (Figure 8). The majority of SNPs led to a significant increase in Hes1 expression, although, p.Ile874= samples expressed significantly lower levels of Hes1 compared to wild-type (WT) controls (Figure 8). Our concluding studies reveal a direct mutagenic effect of smoking on Notch 1 and 2 along with the correlation of specific mutations with signficantly altered expression levels of downstream signaling molecules. To our knowledge this is the first direct evidence linking cigarette exposure to Notch mutations, changes in Notch signaling and ultimately lung tumorigenesis and progression.

The role of NOTCH in tumorigenesis multifaceted and context dependent as evidenced by our results and others demonstrating NOTCH as an oncogene, whereas, other studies in cancers, such as small cell lung cancer, depict NOTCH as a tumor suppressor gene [9–17, 22]. NOTCH signaling plays numerous roles in lung development and interacts with a variety of other signaling pathways, therefore, understanding the cellular and environmental context by which NOTCH functions as an oncogene or tumor suppressor gene is critical to developing effective treatments for specific cancer types.

## MATERIALS AND METHODS

### Construction and transfection of the recombinant pIRES2-EGFP-Numb plasmid

The full-length cDNA sequence of the Numb gene was amplified by polymerase chain reaction (PCR) and inserted into the expression plasmid vector pIRES2-EGFP. Sequence was confirmed by DNA sequencing and the recombinant plasmids were named pIRES2-EGFP-Numb. The recombinant plasmids were transfected into the immortalized human BEP2D cells using Lipofectamine 2000 according to the instructions from the manufacturer (Invitrogen, USA), with the empty plasmid transfection as control.

### Cell cultures and cigarette smoke condensate (CSC) treatment

BEP2D cells were a generous gift from Professor Maoxiang Zhu in Academy of Military Medical Sciences. Cells were routinely cultured in serum free LHC-8 medium at 37°C in a humidified incubator containing 5% CO2. Media was changed every 2 days and cells were subcultured following digestion with 0.25% trypsin/0.02% EDTA in phosphate buffered saline (PBS) every 5 days. CSCs were generated using a HRH-SM120 smoking machine (Huironghe Technology, Beijing, China). The smoke condensates were dissolved in LHC-8 media, at a concentration of 1 cigarette/ml.

### Mouse model of spontaneous tumors

Animal experiments were reviewed and approved by the institutional animal review board at First Affiliated Hospital of Bengbu Medical College and mice were housed in ventilated cages with standard temperature, humidity, exposed to a 12-hourly light/dark cycle and provided with standard diet and water until treatment. A/J mice were used to establish spontaneous tumors model. The mice were put into special smoking machine for smoke exposure experiments (conditions: 50minutes/day, 6days/week, Nicotine 7 mg/m^3^, CO 280ppm, relative humidity 48%±5% and temperature 21°C±1°C). Histological analysis confirmed presence of lung cancer.

### Clinical specimens from heavy smokers and controls

The protocol was approved by the Institutional Review Board of First Affiliated Hospital of Bengbu Medical College and all patients provided informed consent for use of samples in these studies. Patients were screened for being heavy smokers for at least 10 years or having no history of smoking and bronchial mucosa tissue obtained by AFI or NBI bronchoscopic biopsy. mRNA expression levels of Notch1, Notch2, JAG1, JAG2, Hes1, Hey1, Hey2, RBPJ and Numb were determined by quantitative-PCR (qPCR). 50 cases of non-small cell lung cancer (NSCLC) paraffin specimens collected after surgery (29 severe-smokers, 21 never-smokers) were used for the detection of Notch1, Notch2, Hes1 and Numb with Immunohistochemistry (IHC).

Mutations in the coding region of Notch1 and Notch2 were detected in passage 0 (P0), P50, P70 and NSCLC tissue samples of 34 cases by PCR resequencing method. The occurrence frequency of each mutation site between the smoking group and non-smoking group was compared and IHC was performed to measure Hes1 expression in NSCLC tissues with different frequency of mutations.

### MTT assay

BEP2D cells (6×l0^3^) were plated in 96-well plate for 20 hours before incubation with 3-(4,5-dimethylthiazol-2-yl)-2,5-diphenyltetrazolium bromide (MTT) solution (Zhongshan Corp, Beijing, China) for an additional 4 hours. DMSO was then added to the culture wells to solubilize the reactive crystals, and the absorbance at 595 nm was recorded using a 96-well plate reader (Bio-Tek, Vermont, USA).

### Colony formation assay

BEP2D cells were harvested, suspended in LHC-8 medium, and seeded upon a base layer of soft agar at a density of 500 cells per 60mm dish. All experiments were conducted in triplicates. Dishes were maintained at 37°C in humidified incubator and were fed every 3 day. After 6 days, cells were showed by Giemsa staining, and the number of the colony formation was assessed by counting under microscope. CFE (colony form efficiency) (%) = (number of colonies)/(number of inoculation cells) × 100%.

### Annexin V - propidium iodide (PI) staining

1×10^5^ cells were washed and resuspended with PBS. Apoptotic cells were identified by double supravital staining with recombinant fluorescein isothiocyanate-conjugated (FITC) Annexin-V and PI, using the Annexin V-FITC Apoptosis Detection kit (Beyotime Biotech, Shanghai, China) according to the manufacturer’s instructions. Flow cytometric analysis was performed immediately after staining. Data acquisition and analysis were performed in a Becton Dickinson FACS Calibur flow cytometer using CellQuest software.

### Quantitative PCR (qPCR)

Total RNA was isolated from cultured cells or tissues using the TRIzol® Reagent (Invitrogen, San Diego, CA, USA) according to the manufacturer’s instructions. qPCR using SYBR green I was carried out to compare the relative expression of specific mRNAs, as previously described [23]. GAPDH was used as an endogenous control. The comparative CT (ΔΔCT) method was used to calculate target mRNA expression relative to endogenous controls and non-smoking/non-CSC exposed samples.

### Western blot

Total protein from BEP2D cells was extracted using RIPA buffer (Beyotime Biotech, Shanghai, China). Equal concentrations of protein were separated by SDS-PAGE and transferred onto PVDF membranes. After blocking in 3% bovine serum albumin (BSA) solution, membranes were incubated overnight with primary antibodies: rabbit monoclonal anti-Notch1 (1:1000, cell signaling technologies (CST), Shanghai, China), rabbit monoclonal anti-Notch2 (1:1000, CST, Shanghai, China), rabbit monoclonal anti-JAG1 (1:1000, abcam, Shanghai, China), rabbit polyclonal anti-JAG2 (1:1000, Santa Cruz, Shanghai, China), rabbit polyclonal anti-Hes1 (1:1000, abcam, Shanghai, China), rabbit polyclonal anti-Hey1 (1:1000, abcam, Shanghai, China), rabbit polyclonal anti-Hey2 (1:1000, proteintech), rabbit polyclonal anti-DNM1 (1:800, abcam, Shanghai, China), rabbit monoclonal anti-RBPJ (1:800, abcam, Shanghai, China), mouse monoclonal anti-Numb (1:800, abcam, Shanghai, China), rabbit polyclonal anti-GAPDH (1:1000, abcam, Shanghai, China). HRP-conjugated goat anti-rabbit or anti-mouse secondary antibody (Santa Cruz, Shanghai, China) were used at 1:2000. Color development was performed using the Enhanced Chemiluminescence System (Pierce, Rockford, IL, USA). All cases and controls were analyzed in the same experiment, and experiments were performed in triplicates. Optical densities of the bands were analyzed using ImageJ software. For analysis, protein levels were normalized to total protein levels then expressed as a percentage of that in controls.

### Immunofluorescent staining

Cells were grown and differentiated on coverslips coated with 200 μg/ml poly-l-ornithine. Cells were then fixed in 4% formaldehyde for 20 minutes and stored in PBS. Membranes were permeabilized with 0.25% Triton X-100, and nonspecific binding was blocked with 1% BSA for 30 minutes. Cells were incubated overnight with primary antibodies: rabbit monoclonal anti-Notch2 (1:1600, CST, Shanghai, China), mouse monoclonal anti-Numb (1:1000, abcam, Shanghai, China). Cells were then incubated for 2 hours with goat anti-mouse or rabbit CY3 554–conjugated secondary antibodies (1:400). coverslips were then incubated with DAPI nucleic acid stain (l μg/ml) for 10 minutes and mounted with glycerin.

### Immunohistochemical staining

The paraffin embedded sections (thickness, 4μm) were de paraffinized completely. To retrieve the antigens, the slides were immersed in citric acid buffer (10 mmol/L of citrate sodium and 10 mmol/L of citric acid) and boiled in a microwave oven at 92–98°C for 15 minutes. The sections were cooled to room temperature and sequentially incubated at room temperature with 3% H2O2 in methanol for 15 minutes to quench endogenous peroxidase and in normal blocking serum for 30 minutes. The slides were then incubated with mouse monoclonal anti-XIAP (1:300, abcam, Shanghai, China), rabbit monoclonal anti-Survivin (1:300, abcam, Shanghai, China), mouse monoclonal anti-TTF-1 (1:500, abcam, Shanghai, China), mouse monoclonal anti-P63 (1:500, Santa Cruz, Shanghai, China), mouse monoclonal anti-CK5/6 (1:500, Santa Cruz, Shanghai, China), rabbit monoclonal anti-Notch1 (1:200, CST, Shanghai, China), rabbit monoclonal anti-Notch2 (1:200, CST, Shanghai, China) or mouse monoclonal anti-Numb (1:250, abcam, Shanghai, China) at 4°C overnight and stained with DAB. Intervening PBS washes were performed after incubation when necessary.

### PCR direct sequencing

The coding sequence region of Notch1 and Notch2 were amplified by PCR. The PCR products were purified with 96 well purification plates (Millipore, Shanghai, China). Sanger sequencing was performed using Thermo Sequenase Dye Primer Manual Cycle Sequencing kit (ThermoFisher Scientific, MA, USA). Sequencing reaction volumes contained 2μL Mix (Bigdye3.1, 5×sequencing buffer, H2O), 2μL purified PCR products, 1μL primers (5 mmol/L). Sequencing conditions were: 95°C15seconds → (95°C15seconds→50°C 5seconds→60°C90seconds) × 35cycles. Specific SNPs identified in Notch1 and 2 are outlined in Tables 1 and 2, respectively.

**Table 1:** The correlation of smoking and Notch1 coding sequcence mutation

Location	Sequence variation	SNP ID	Amino acid variation	Mutation sequence	Frequency (%) in smoker and never smoker
exon27	c.5094C>T	rs10521	p.Asp1698=	GTGCCACCGA[T/T]GTGGCCGCAT	Smoker:15.8%^*^ Non-smoker:6.7%
exon14	c.2265T>C	rs2229971	p.Asn755=	TCAACAACAA[C/C]GAGTGTGAAT	Smoker:57.9%^*^ Non-smoker:60%
exon34	c.6555C>T	rs2229974	p.Asp2185=	AGTCCCAGGA[T/T]GGCAAGGGCT	Smoker:42.1%^*^ Non-smoker:46.7%
exon34	c.6648G>A	rs3812596	p.Pro2216=	TGGCCTCGCC[A/G]CCACTGCTGC	Smoker:10.5%^*^ Non-smoker:6.7%
exon3	c.312T>C	rs4489420	p.Asn104=	CCCTGGACAA[C/C]GCCTGCCTCA	Smoker:15.8%^*^ Non-smoker:6.7%

^*^P<0.05 Smoker vs Never-smoker (Fisher’s Exact Test).

**Table 2:** The correlation of smoking and Notch2 coding sequcence mutation

Location	Sequence variation	SNP ID	Amino acid variation	Mutation sequence	Frequency (%) in smoker and never smoker
exon1	c.57C>G	rs11810554	p.Cys19=	GGCTGTGCTG[C/G] GCGGCCCCCG	Smoker:10.5%^*^ Non-smoker:13.3%
exon4	c.710G>A	rs146498360	p.Arg237Gln	GGCACCTGTC[A/G] GCAGACTGGT	Smoker:21.0%^*^ Non-smoker:0
exon1	c.61G>A	rs2603926	p.Ala21Thr	GTGCTGCGCG[A/A] CCCCCGCGCA	Smoker:31.6%^*^ Non-smoker:6.7%
exon28	c.5065A>T	rs60854092	p.Ile1689Phe	TGTTGTCATC[A/T] TTCTGTTTAT	Smoker:10.5%^*^ Non-smoker:0
exon34	c.7042T>C	rs61734328	p.Leu2348=	AATGGCCCGT[C/T] TGCCCAGTGT	Smoker:10.5%^*^ Non-smoker:0
exon20	c.3234C>T	rs7543643	p.Cys1078=	AAGGTACTTG[C/T] GTTCAGAAAA	Smoker:10.5%^*^ Non-smoker:13.3%
exon4	c.572A>T	Novel	p.His191Leu	ATTCCAGGAC[A/T] CTGCCAGCAT	Smoker:10.5%^*^ Non-smoker:0
exon17	c.2622T>C	Novel	p.Ile874=	CCATTGACAT[C/C] GACGAGTGTA	Smoker:10.5%^*^ Non-smoker:0
exon18	c.2882G>A	Novel	p.Gly961Glu	TGTAAGAATG[A/A] AGGGACCTGC	Smoker:15.8%^*^ Non-smoker:0

^*^P<0.05 Smoker vs Never-smoker (Fisher’s Exact Test).

### Statistical analysis

Results are expressed as mean ± standard deviation (SD). Statistical analysis was performed using analysis of variance (ANOVA), Fisher’s Exact Test and the Student’s t-test to test for significant differences. Analyses were conducted using SPSS 10.0 program (Chicago, USA). P values < 0.05 were considered significant.

## Abbreviations

JAG1: jagged1
Hey1: hairy/Enhancer-Of-Split Related With YRPW Motif 1
Hey2: hairy/Enhancer-Of-Split Related With YRPW Motif 2
JAG2: Numb, jagged2
Hes1: Notch2, hairy and enhancer of split 1
DNM1: Dynamin-1
RBPJ: recombining binding protein suppressor of hairless
qPCR: quantitative polymerase chain reaction
NSCLC: non-small cell lung cancer
FITC: fluorescein isothiocyanate-conjugated
PI: propidium iodide
TTF-1: Transcription Termination Factor 1
XIAP: X-linked inhibitor of apoptosis
RNA: ribonucleic acid
GAPDH: Glyceraldehyde 3-phosphate dehydrogenase
IHC: immunohistochemistry
